# Postoperative improvement in DASH score, clinical findings, and nerve conduction velocity in patients with cubital tunnel syndrome

**DOI:** 10.1038/srep27497

**Published:** 2016-06-06

**Authors:** Yoshikazu Ido, Shigeharu Uchiyama, Koichi Nakamura, Toshiro Itsubo, Masanori Hayashi, Yukihiko Hata, Toshihiko Imaeda, Hiroyuki Kato

**Affiliations:** 1 Rehabilitation Center, Shinshu University Hospital, Matsumoto, Japan; 2 Department of Orthopaedic Surgery, Shinshu University School of Medicine, Matsumoto, Japan; 3 Department of Orthopaedic Surgery, North Alps Medical Center Azumi Hospital, Ikeda, Japan; 4 Sports Medicine Center, Aizawa Hospital, Matsumoto, Japan; 5 Department of Food and Nutritional Environment, Kinjo Gakuin University, Nagoya, Japan

## Abstract

We investigated a recovery pattern in subjective and objective measures among 52 patients with cubital tunnel syndrome after anterior subcutaneous transposition of the ulnar nerve. Disabilities of the Arm, Shoulder and Hand (DASH) score (primary outcome), numbness score, grip and pinch strength, Semmes-Weinstein (SW) score, static 2-point discrimination (2PD) score, and motor conduction velocity (MCV) stage were examined preoperatively and 1, 3, 6, 12, and ≥24 months postoperatively. Statistical analyses were conducted to evaluate how each variable improved after surgery. A linear mixed-effects model was used for continuous variables (DASH score, numbness, grip and pinch strength), and a proportional odds model was used for categorical variables (SW and 2PD tests and MCV stages). DASH score significantly improved by 6 months. Significant recovery in numbness and SW test scores occurred at 1 month. Grip and pinch strength, 2PD test scores, and MCV stage improved by 3 months. DASH scores and numbness recovered regardless of age, sex, or disease severity. It was still unclear if both subjective and objective measures improved beyond 1-year postoperatively. These data are helpful for predicting postoperative recovery patterns and tend to be most important for patients prior to surgery.

Cubital tunnel syndrome (CubTS) is the second most common entrapment neuropathy in the upper extremity (following carpal tunnel syndrome), and the number of surgical treatments available for this condition is increasing^1^. Although several surgical techniques have been advocated in the past, none of the procedures was superior to the others. This is partly due to a lack of reliable, reproducible, and valid outcome measures^2^,^3^. Thus, a standardized assessment protocol for CubTS is necessary for comparing these procedures. Ideally, optimal surgical techniques should be applied to each patient based on nerve palsy pathophysiology.

It would also be very helpful to predict when postoperative improvement among those parameters occurs and the length of improvement; this tends to be most important for patients prior to surgery. In order to meet such expectations, multiple time-point surveys post-surgery are necessary.

Recently, recovery patterns among various evaluation variables for patients with CubTS, observed 1-year post-*in situ* decompression surgery, have been reported^4^. However, the distance between the entrapment point of the ulnar nerve and CubTS target organs is much longer than with carpal tunnel syndrome; thus, functional recovery may be delayed and take even longer than a year^5^.

Therefore, we investigated recovery patterns in subjective and objective measures, including nerve conduction studies, 2 or more years after an anterior subcutaneous transposition of the ulnar nerve. This surgical procedure was chosen because it is one of the most representative procedures for CubTS. Our primary outcome included responses on the Disabilities of the Arm, Shoulder, and Hand (DASH) score^6^, since it is widely used for upper extremity disorders.

## Methods

A CubTS diagnosis was made for 105 consecutive patients in our institution from June 2006 to August 2012. We performed 91 surgeries for 91 patients with CubTS. The inclusion criteria included CubTS accompanied by osteoarthritic changes of the elbow joint, a sensory or motor deficit confirmed by a physical examination and nerve conduction studies, and being treated with an anterior subcutaneous transposition of the ulnar nerve. We included patients with elbow osteoarthritis since CubTS is associated with this condition in Japan^7^,^8^. Elbow osteoarthritis was evaluated by measuring range of motion and plain radiographs in anteroposterior and lateral views. Elbows with a Kellgren-Lawrence classification^9^ grade = 1 or higher were regarded as osteoarthritic. In an elbow osteoarthritis case, the ulnar nerve runs more posteriorly from the center of elbow rotation. This is because the sulcus nervi ulnaris becomes shallow due to underlying osteophyte formation, as opposed to the absence of osteophyte formation. This could put the nerve under greater compression during elbow flexion. In our practice, anterior subcutaneous transposition of the ulnar nerve is indicated for this condition.

**Figure 1:**
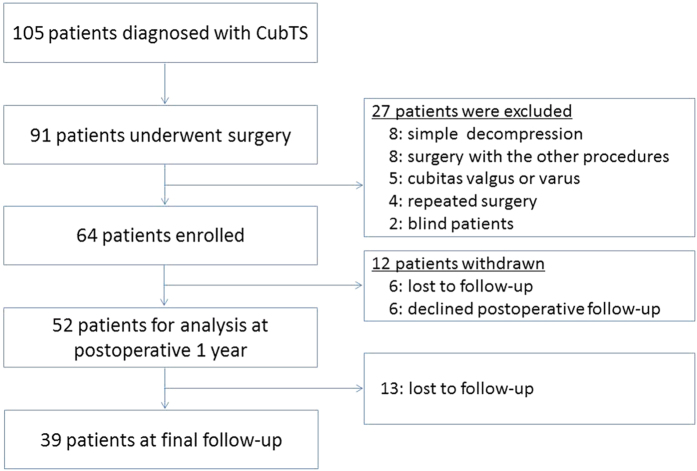


Exclusion criteria were a history of ulnar nerve decompression, cubital tunnel syndrome that was idiopathic or caused by conditions other than osteoarthritis (including contusion, dislocation, and fracture), or concomitant cervical lesions. When symptoms or physical findings led to a suspicion of cervical lesions, such as C6-C8 radiculopathy or myelopathy, magnetic resonance imaging of the cervical spine was performed. In such cases, only patients without lesions in the cervical spine were included. Patients with tardy ulnar nerve palsy associated with cubitus valgus or varus were also excluded, as were patients who underwent concomitant procedures such as tendon transfer or carpal tunnel release, in addition to the ulnar nerve transposition. As a result, 27 patients were excluded, and 64 patients were enrolled (Fig. 1). All patients had osteoarthritic changes in the elbow, with a decreased range of motion (flexion: 122 ± 8.1°, extension: −17 ± 8.7°) and without severe pain. Disease severity was determined with the McGowan classification^10^ (grade I–III). Patient demographic data prior to surgery are shown in Table 1.

This is a retrospective study. Shinshu University School of Medicine’s ethical committee approved the study protocol, and written informed consent was obtained from all patients prior to surgery. The methods were carried out in accordance with the approved guidelines.

### Preoperative and postoperative clinical evaluation

We obtained the following clinical parameters for our data analyses. Patients were asked to complete the Japanese version of the validated patient-oriented questionnaire of the DASH score^11^. Numbness in the little finger was quantified from 0 (no numbness) to 10 (the most severe numbness ever experienced), using a visual analogue scale (VAS). Nerve conduction velocities (NCVs) were examined according to the American Association of Electrodiagnostic Medicine guidelines^12^, using Neuropack M1 (Nihon Kohden, Tokyo, Japan). Ulnar antidromic sensory and orthodromic motor stimulation at the distal and proximal forearms, and above the elbow, was performed. Compound muscle action potentials (CMAPs) and sensory nerve action potentials (SNAP) were recorded, and motor conduction velocities (MCVs) across the elbow and sensory nerve conduction velocities (SCVs) were calculated. Room temperature was kept at 25 °C. Skin temperature was over 31°C. Since we could not detect SNAPs for most of the patients, evaluation was performed by looking only at MCV, which was classified as follows: normal (or 1), normal MCV (≥50 m/s); moderate (or 2), slow MCV; severe (or 3), absent CMAP. CMAP was not always detected by electrical stimulation. Therefore, since MCV could not be calculated for each case, a grade classification was used. Grip strength was measured using a Jamar dynamometer (Sammons Preston Rolyan, Bolingbrook, IL), and side pinch strength was measured using a pinch gauge (Sakai Medical Co. Ltd., Tokyo, Japan). Measurements were repeated 3 times, and the mean value was used. Using a classification system developed by Gelberman *et al*., we also evaluated atrophy of the abductor digiti minimi muscle^13^.

For sensory evaluation, the Semmes-Weinstein (SW) monofilament test (a nylon monofilament set; Sakai Medical Co. Ltd.) and the static 2-point discrimination (2PD) test (Sakai Medical) were used to obtain measurements from the little and index fingers. Interpretation of the SW and 2PD results was based on international criteria^14^,^15^: for the SW test—normal (or stage 1), ≤ 2.83; diminished touch (stage 2), 3.22–3.61; diminished protective sensation (stage 3), 3.84–4.31; loss of sensation (stage 4), 4.56–6.65; untestable (stage 5), > 6.65: for the 2PD test—normal (stage 1), ≤ 5 mm; fair (stage 2), 6–10 mm; poor (stage 3), 11–15 mm; untestable (stage 4), > 15 mm.

DASH score, numbness score, grip and pinch strength, Semmes-Weinstein (SW) score, static 2-point discrimination (2PD) score, and motor conduction velocity (MCV) stage were examined preoperatively and 1, 3, 6, 12, and ≥24 months postoperatively.

### Surgical procedure

Surgery was performed under general anesthesia. A curved skin incision, approximately 10–13 cm long, was centered over (and posterior) to the medial epicondyle of the humerus. The ulnar nerve was identified just proximal to the entrance of the cubital tunnel, and the cubital tunnel retinaculum was incised. The deep forearm fascia over the ulnar nerve was also incised. The arcade of Struthers and the medial intermuscular septum of the upper arm were removed to allow anterior displacement of the ulnar nerve without tension or kinking. The nerve was transposed anteriorly with nutrient vessels, if possible. The adipofascial fat flap from the skin was sutured to the medial epicondyle to keep the ulnar nerve over the flexor muscles’ origin, not back to the cubital tunnel. The elbow was splinted for the first 2 postoperative days. Unrestricted use of the involved hand, depending on the patient’s comfort level around the elbow, was then allowed.

### Statistical analyses

Statistical analyses were conducted to evaluate how each variable improved after surgery. A linear mixed-effects model was used for continuous variables (DASH score, numbness, grip and pinch strength), and a proportional odds model was used for categorical variables (SW and 2PD tests, and MCV stages). Covariates comprised age, sex, and preoperative disease severity (McGowan grade) in both models. Age was divided into 4 categories: 1, 50–59; 2, 60–69; 3, 70–79; 4, ≥80 years. Any interaction variables among the covariates were included in both models. All the patients were included in these models.

To determine when significant improvement in each parameter first occurred compared with the preoperative value, and to determine if any improvement was found between the 12-month and final follow-ups, multiple-comparison tests using linear mixed models (with estimated marginal means) or proportional odds model were employed for each variable. A significance level for statistical test of coefficients in each model was set by Bonferroni’s correction. For our power analysis, the minimal, clinically important difference (MCID) in DASH scores (12 months postoperatively) was 7 points (standard deviation: 17.6)^4^,^16^. Thus, at least 52 patients were needed to detect a significant difference in DASH scores with 80% power (a 2-tailed, paired t-test; α = 0.05)^17^. The MCIDs of grip strength and pinch strength were 2.4 kg and 0.54 kg, respectively^18^. In this case, at least 40 and 47 patients were needed to detect a significant difference in grip strength and pinch strength, with 80% power (a 2-tailed, paired t-test; α = 0.05), respectively.

A linear mixed-effects model was conducted using SPSS version 22 (SPSS Inc., Chicago, IL), and a proportional odds model was conducted using R version 3.2.3 (R Foundation for Statistical Computing, Vienna, Austria). All statistical significances were set at p < 0.05.

Finally, for each variable, the number of patients whose data improved to normal values (during the 2+ follow-up years) was calculated. Changes in McGowan stage and abductor digiti minimi muscle atrophy grade were also described during follow up.

## Results

Data for each variable, as a function of postoperative time, are listed in Tables 2 and 3. Fifty-two patients were followed up to at least one year post-surgery, and 13 patients (25%) did not visit our hospital at the final follow-up. Thus, 39 patients were followed for 2 years or more (average: 34.8 months; range: 24–78 months) (Fig. 1).

### Improvement among each variable

**Figure 2:**
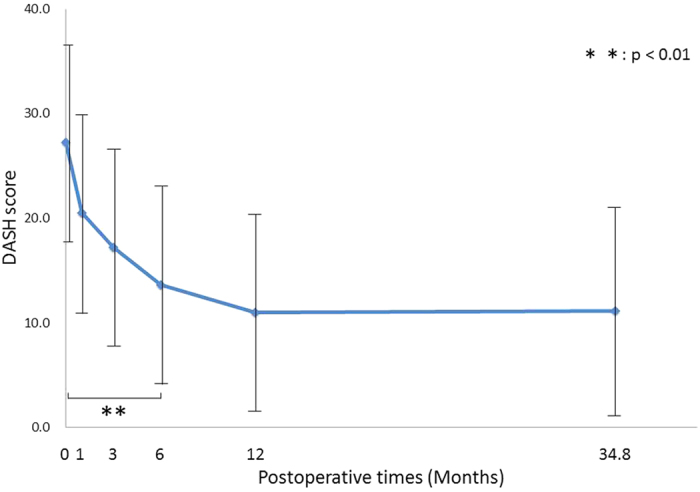
Postoperative changes in DASH score. Changes in the estimated marginal means for the Disabilities of the Arm, Shoulder, and Hand (DASH) scores as a function of postoperative time. The DASH score significantly improved 6 months postoperatively, regardless of age, sex, or disease severity. There was no significant difference between the 12-month and final follow-ups.

DASH scores significantly improved 6 months postoperatively, regardless of age, sex, or disease severity (p = 0.003). This improvement was more than a minimal clinically important difference (Fig. 2). There was no significant difference in DASH scores between the 12-month and final follow-ups (p = 0.40).

**Figure 3:**
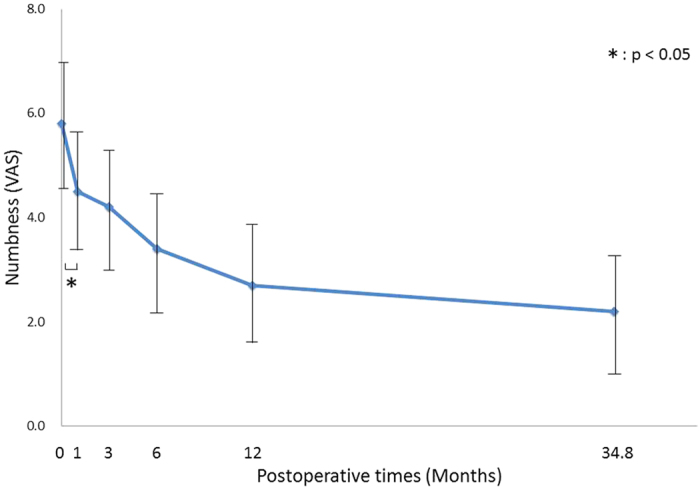
Postoperative changes in Numbness. Changes in the estimated marginal means for numbness visual analogue scale (VAS) scores as a function of postoperative time. Numbness significantly improved 1 month postoperatively, regardless of age, sex, or disease severity, and there was no significant difference between the 12-month and final follow-ups.

Numbness VAS scores significantly improved at 1 month, regardless of age, sex, or disease severity (p = 0.01). Furthermore, there was no significant difference between the 12-month and final follow-ups (p = 0.25) (Fig. 3).

**Figure 4:**
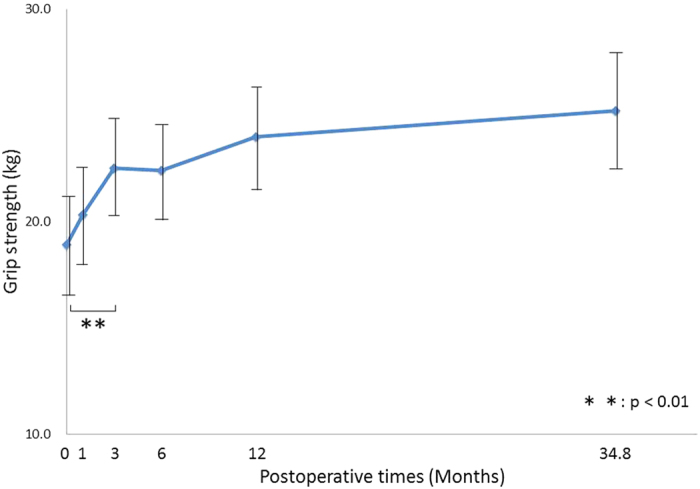
Postoperative changes in Grip strength. Changes in the estimated marginal means for grip strength as a function of postoperative time. Grip strength significantly improved 3 months postoperatively, and there was no significant difference between the 12-month and final follow-ups.

Grip strength significantly improved at 3 months (p < 0.001), and there was no significant difference between the 12-month and final follow-ups (p = 0.40). Age, sex, and disease severity significantly affected grip strength recovery; elderly patients had less grip strength than younger patients; men had an average grip strength of greater than 10.1 kg (95% CI: 5.8, 14.5) compared to women; and preoperative McGowan grade II patients had an average of 4.3 kg (95% CI: 1.4, 7.2) greater strength than grade III patients (Fig. 4).

**Figure 5:**
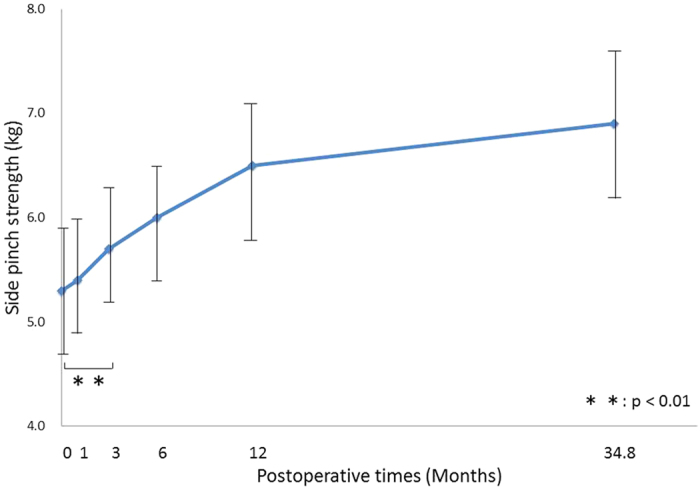
Postoperative changes in Side pinch strength. Changes in the estimated marginal means for pinch strength as a function of postoperative time. Pinch strength significantly improved 3 months postoperatively, regardless of age, and there was no significant difference between the 12-month and final follow-ups.

Pinch strength significantly improved at 3 months (p = 0.006), regardless of age, and there was no significant difference between the 12-month and final follow-ups (p = 0.40) (Fig. 5). However, sex and disease severity significantly affected strength; men had an average pinch strength 2.2 kg (95% CI: 1.1, 3.3) greater than women; and preoperative McGowan grade II patients had a 1.4 kg (95% CI: 0.7, 2.2) greater strength than grade III patients.

SW score was significantly improved at 1 month, regardless of age (p = 0.017), and there was no significant difference between the 12-month and final follow-ups (p = 0.63). Sex and Disease severity significantly affected recovery: female tended to be better than male (p < 0.001), and grade II was always better than grade III (p < 0.001).

The 2PD score significantly improved at 3 months, regardless of age and sex (p < 0.01) and did not change thereafter (p = 0.72). Disease severity significantly affected recovery: grade II was better than grade III (p < 0.001).

MCV stage significantly improved at 3 months (p = 0.018), regardless of age and sex; there was no significant difference between the 12-month and final follow-ups (p = 0.31). Disease severity significantly affected MCV stage recovery: grade II was always better than grade III (p < 0.001).

### Overall results at the final follow-up

After an average of 34.8 months, all variables significantly improved from their preoperative levels, but not all recovered to within a normal range. Numbness resolved in 33.3% of patients, and SW and 2PD scores (and MCV criteria) were normal in 20.5%, 35.9%, and 44.8% of patients, respectively. Preoperatively, no patients were classified as McGowan grade I, 25 patients were grade II (48.1%), and 27 were grade III (51.9%). At the final follow-up, 5 patients were grade I (12.8%), 25 patients were grade II (64.1), and 9 patients were grade III (23.1%). Preoperatively, atrophy of the muscle was absent in 1 (1.9%), mild to moderate in 33 (63.5%), and severe in 18 (34.6%) patients. At the final follow-up, no atrophy was observed in 10 (25.6%), mild to moderate in 20 (51.3%), and severe in 9 (23.1%) patients. There was only 1 patient out of 64 at enrollment whose chief complaint was elbow pain due to osteoarthritis, as well as symptoms caused by ulnar nerve palsy. This patient did not need any surgical interventions for the elbow pain, such as osteophyte debridement, because the pain was tolerable. We prescribed nonsteroidal anti-inflammatory drugs to the patients, and pain levels did not increase at the final evaluation.

## Discussion

We investigated recovery patterns (for ≥2 years) among various variables, including MCV stage, for patients with CubTS after an anterior subcutaneous ulnar nerve transposition. Improvement in DASH scores for CubTS, after various procedures, has been reported^4^,^17^,^19^,^20^.

Giladi *et al*.^4^ reported a recovery pattern in DASH scores after surgery. According to their results, significant improvement in DASH scores was observed and reached a plateau 3 months postoperatively, with no significant changes seen within 1 year after surgery. Ebersole *et al*.^20^ observed a similar recovery trend with moderate changes in DASH scores beyond the 3-month follow-up, after anterior transmuscular transposition of the ulnar nerve. In our study, significant improvement was observed 6 months postoperatively, with smaller improvements observed thereafter regardless of age, sex, or disease severity. Thus, we cannot deny the possibility that DASH scores improve more slowly after anterior subcutaneous transposition of the ulnar nerve than after simple decompression or anterior transmuscular transposition. It should be noted, however, that the patient characteristics, follow up rate, and study design differ among the studies. Our data demonstrated no significant differences between this first postoperative year and final follow-up. It is likely that definitive conclusions could not be drawn due to a lack of statistical power resulting from the loss of 13 patients (25%) during the follow-ups.

The present findings revealed that numbness recovered first, followed by grip and pinch strength, consistent with a previous report^4^. Recovery of grip and pinch strength was affected by preoperative disease severity and sex; however, recovery in numbness VAS scores was not. It is unclear why there were discrepancies in recovery between symptoms (numbness) and functions (grip and pinch strength). Possibly, through decompression and transposition of the nerve, intraneural blood flow could be restored soon after surgery, regardless of disease severity. This could help alleviate the tingling sensations or numbness resulting from nerve ischemia; however, recovery to the highly degenerated axons (with atrophied muscles) could take longer before meaningful strength was restored. It is well known that even if a regenerating motor axon reaches a denervated muscle, muscle function does not recover as quickly. This is because it takes time to re-establish neuromuscular junction, as well as functional reflex activity upon nerve stimulation^21^. This was consistent with the recovery in grip strength observed in the present study, which increased significantly.

SW score tended to be better in female than male, although 2PD score had not affected by sex in present study. Weinstein reported that women demonstrated significantly greater pressure sensitivity at little finger than men; however, 2PD had not significant difference in same area^22^. This fact accords and supports our results.

Most previous CubTS studies measured NCVs at only 1 time point after surgery^23^,^24^,^25^, making determination of recovery patterns difficult. Our study found that SCV was rarely measurable before surgery, and SCV recovery was hard to detect after surgery, even if MCV recovered. These findings could be explained as follows: generally, SCV is determined based on the fastest fibers inside the peripheral nerve, such as Aα fibers, which conduct touch or pressure signals from tactile receptors in the skin. When the nerve is subject to chronic compression, the SNAP amplitude is very sensitive to axonal loss because of the poor supportive effect of collateral re-innervation. After surgery, some sensory fibers could recover, but perhaps not in sufficient number to detect SNAPs. In our study, significant MCV stage recovery was observed 3 months postoperatively, regardless of age or sex, and no significant difference was detected between the 12-month and final follow-ups. This suggested MCV stage recovery was unlikely beyond 1 year post-surgery. Nabhan *et al*.^26^ measured MCV of the ulnar nerve across the elbow before, and at 3 and 9 months after, simple decompression or anterior subcutaneous transposition surgery for comparison. They found that both procedures demonstrated a similar recovery pattern: significant recovery at 3 months that seemed to reach a plateau by 9 months postoperatively. Although their patients were at a relatively early disease stage, recovery of MCV seemed as fast as it was in our patients. MCV stage improvement in our study is somewhat inconsistent with a previous report from Matsuzaki *et al*.^5^ in which they observed improvement in NCV beyond 2 years post-surgery. This inconsistency may be partly attributed to differences in how NCVs were evaluated; our MCV classification into categories is not as sensitive as raw MCV or SCV values, if they are measurable.

Our study has some notable strengths. For instance, a long follow-up period allowed for comparisons between a 12-month and proceeding follow-up periods, and a linear mixed-effects model allowed us to calculate pattern changes among all of our variables. This aided our clear results interpretation. Nevertheless, several limitations should also be noted. A total of 13 patients (25.0%) were lost during follow-up after 12 months. Thus, analyses with the DASH score, grip strength, and pinch strength between the 12-month and final follow-ups were underpowered. Improved pinch strength at 3 months may be clinically insignificant, as strength was less than MCID. Since MCID was not determined based on numbness VAS, we did not have sufficient power to conduct this analysis. Since most patients with CubTS also have osteoarthritic changes to the elbow in Japan, we did not include 8 patients with idiopathic CubTS, all of whom underwent *in situ* decompression of the ulnar nerve. A recent trend in surgical procedures has shifted from an anterior transposition to *in situ* decompression^1^. Thus, our patient spectrum differed from that of previous studies; therefore, generalizability of the present findings beyond patients with ulnar nerve compression associated with elbow osteoarthritis is limited. Moreover, our findings and conclusions cannot be guaranteed to apply to other ethnic groups or geographical regions. We also did not use the Michigan Hand Questionnaire, which tends to be more sensitive than a DASH score^4^; this might have been one reason we detected slow recovery among our patients. Additionally, we could not definitively determine when plateaus were reached among our variables since follow-ups did not extend beyond an average of 34.8 months. Finally, since the number of female patients was small, the effect of sex on the studied variables should be interpreted with caution.

In summary, DASH score significantly improved by 6 months after surgery. Significant recovery in numbness and SW test scores occurred by 1 month. Grip and pinch strength, 2PD test scores, and MCV stage improved by 3 months. DASH scores and numbness recovered regardless of age, sex, or disease severity. It is unclear whether both subjective and objective measures improved beyond the first postoperative year. Nevertheless, the present findings are helpful for predicting postoperative recovery patterns when preparing patients for surgery.

## Additional Information

**How to cite this article**: Ido, Y. *et al*. Postoperative improvement in DASH score, clinical findings, and nerve conduction velocity in patients with cubital tunnel syndrome. *Sci. Rep.* **6**, 27497; doi: 10.1038/srep27497 (2016).

## Figures and Tables

**Table 1:** Demographic data.

			Value
Age, yrs.	Mean (SD)		67.3 (8.8)
	Range		51–83
Sex	Male		44 (84.6%)
	Female		8 (15.4%)
McGowan Grade	I	Mild	0 (0%)
	II	Intermediate	25 (48.1%)
	III	Severe	27 (51.9%)

**Table 2:** DASH, clinical findings, and neurophysiological variables as a function of postoperative time.

	Preoperatively	n	1 Month	n	3 Months	n
DASH score	27.2 (17.8, 36.6)	52	20.5 (11.0, 30.0)	48	17.2 (7.8, 26.6)	51
Numbness, VAS	5.8 (4.6, 7.0)	52	4.5 (3.4, 5.7)	46	4.2 (3.0, 5.3)	49
No numbness (score of zero)	0.0%	0	13.0%	6	14.3%	7
Grip strength, kg	18.9 (16.6, 21.2)	52	20.3 (18.0, 22.6)	49	22.5 (20.3, 24.8)	51
Key pinch, kg	5.3 (4.7, 5.9)	52	5.4 (4.9, 6.0)	49	5.7 (5.2, 6.3)	52
SW		51		47		50
Normal	5.9%	3	14.9%	7	18.0%	9
Diminished light touch	13.7%	7	25.5%	12	24.0%	12
Diminished protective sensation	51.0%	26	38.3%	18	32.0%	16
Loss of protective sensation	25.5%	13	21.3%	10	26.0%	13
Untestable	3.9%	2	0.0%	0	0.0%	0
2PD		51		47		51
Normal	7.8%	4	10.6%	5	17.6%	9
Fair	21.6%	11	29.8%	14	37.3%	19
Poor	17.6%	9	17.0%	8	15.7%	8
Untestable	52.9%	27	42.6%	20	29.4%	15
MCV		51		44		45
Normal	9.8%	5	22.7%	10	24.4%	11
Delay	72.5%	37	63.6%	28	66.7%	30
Not detected	17.6%	9	13.6%	6	8.9%	4
	**6 Months**	**n**	**12 Months**	**n**	**2 Years or more**	**n**
DASH score	13.6 (4.2, 23.0)	52	11.0 (1.6, 20.4)	51	11.1 (1.1, 21.2)	39
Numbness, VAS	3.4 (2.2, 4.5)	50	2.7 (1.6, 3.9)	51	2.2 (1.0, 3.3)	39
No numbness (score of zero)	14.0%	7	21.6%	11	33.3%	13
Grip strength, kg	22.4 (20.1, 24.7)	52	24.0 (21.6, 26.3)	52	25.2 (22.6, 27.9)	39
Key pinch, kg	6.0 (5.4, 6.5)	52	6.5 (5.8, 7.1)	52	6.9 (6.2, 7.6)	39
SW		52		52		39
Normal	21.2%	11	19.2%	10	20.5%	8
Diminished light touch	30.8%	16	44.2%	23	43.6%	17
Diminished protective sensation	30.8%	16	34.6%	18	33.3%	13
Loss of protective sensation	17.3%	9	1.9%	1	2.6%	1
Untestable	0.0%	0	0.0%	0	0.0%	0
2PD		52		52		39
Normal	21.2%	11	30.8%	16	35.9%	14
Fair	42.3%	22	50.0%	26	41.0%	16
Poor	25.0%	13	13.5%	7	10.3%	4
Untestable	11.5%	6	5.8%	3	12.8%	5
MCV		44		49		29
Normal	27.3%	12	36.7%	18	44.8%	13
Delay	65.9%	29	59.2%	29	51.7%	15
Not detected	6.8%	3	4.1%	2	3.4%	1

Data for DASH, numbness, grip strength, and pinch strength are expressed as estimated marginal means. Each 95% confidence interval is shown in the bracket. DASH: Disabilities of the Arm, Shoulder, and Hand questionnaire, VAS: Visual Analogue Scale, SW: Semmes-Weinstein monofilament test, 2PD: 2-point discrimination test, MCV: motor conduction velocity.

**Table 3:** Score changes between preoperative status and each follow up.

	1 Month	3 Months	6 Months	12 Months	2 Years or more
DASH score	−6.6 (5.0, −18.3)	−10.0 (1.5, −21.5)	−13.6 (−2.1, −25.0)	−16.2 (−4.7, −27.6)	−16.1 (−3.5, −28.6)
Numbness, VAS	−1.3 (−0.1, −2.4)	−1.6 (−0.5, −2.8)	−2.4 (−1.4, −3.5)	−3.1 (−2.0, −4.1)	−3.6 (−2.5, −4.8)
Grip strength, kg	1.4 (−0.4, 3.1)	3.6 (1.9, 5.4)	3.5 (1.6, 5.4)	5.1 (3.1, 7.1)	6.3 (3.6, 9.0)
Key pinch, kg	0.1 (−0.3, 0.5)	0.4 (0.1, 0.8)	0.7 (0.2, 1.1)	1.1 (0.5, 1.7)	1.6 (0.9, 2.3)

Changes in DASH, numbness, grip strength, and pinch strength between the preoperative status and each follow up. Each 95% confidence interval is shown in the bracket. DASH: Disabilities of the Arm, Shoulder, and Hand questionnaire, VAS: Visual Analogue Scale.
